# Effect of noise sensitivity on psychophysiological response through monoscopic 360 video and stereoscopic sound environment experience: a randomized control trial

**DOI:** 10.1038/s41598-022-08374-y

**Published:** 2022-03-16

**Authors:** Hyun In Jo, Kounseok Lee, Jin Yong Jeon

**Affiliations:** 1 Department of Architectural Engineering, Hanyang University, Seoul, 04763 Korea; 2 Department of Psychiatry, Hanyang University Medical Center, Seoul, 04763 Korea; 3 Department of Medical and Digital Engineering, Hanyang University, Seoul, 04763 Korea

**Keywords:** Environmental sciences, Health care

## Abstract

Noise sensitivity is a crucial factor affecting subjective psychophysiological responses to the acoustic environment of various indoor and outdoor spaces. This study examines how noise sensitivity or hyperacusis affects the recovery of emotional and autonomic nervous system (ANS) responses when experiencing various monoscopic 360 video and stereoscopic sound environments (urban and natural) that represent the actual environment. A total of 60 general participants with mild depression, stress, and anxiety were examined using a survey to investigate individual characteristics, including noise sensitivity, and K-means clustering was used to classify them into sensitivity groups. Emotional and physiological responses were measured using the Korean edition of Profile of Mood States and by assessing heart rate variability, respectively. Overall, the emotional recovery effect was greater in the natural than the urban environment, and the homeostatic mechanism of the ANS was better maintained, thereby increasing stress resistance. Noise sensitivity did not have considerable effect on psychophysiological recovery in the natural environment, but had a significant effect on emotional response in the urban environment. This can be used as basic data in seeking customized emotional recovery for individuals using monoscopic 360 video and stereoscopic sound technology in the future.

## Introduction

In general, groups with high noise sensitivity are likely to show greater annoyance to noises that occur inside and outside various spaces^1^. Noise sensitivity refers to the subjective response to noises and is considered to be an individual personality trait^2^. Individual responses to noises may vary depending on non-acoustical factors such as individual personalities, attitudes toward noises, previous experiences, and exposure to the noise environment, and acoustical factors such as sound pressure, noise level, and frequency characteristics^3^. Therefore, to explain noise sensitivity, it is necessary to consider the characteristics of the noise itself and the various non-acoustical factors that affect individual responses.

There are various subjectively unpleasant noise sources such as a door slamming, phone ringing, water running, cooking sounds, and voices. Some of the most common noises include traffic noises, vacuum cleaners, kitchenware and workshop noises, drilling, dishes clanking, and children yelling^4,5^. However, some people also experience earache, dizziness, anxiety, tension, startle response, and panic attacks due to common sound stimuli.

These individual characteristics related to noise sensitivity can be explained by hyperacusis, commonly defined as “a disorder that makes it hard to deal with everyday sounds that others generally do not find uncomfortable”^6^. It refers to a diminished sound tolerance from being considerably more loudness-dependent than others due to oversensitive hearing. Thus, the loudness discomfort level of people with hyperacusis is lower than normal^6^.

Hyperacusis also refers to hyperacute or super-normal hearing or a lowered hearing threshold. However, this is extremely rare in clinical practice and is observed when the superior semicircular canal dehiscence exposes a negative bone-conduction hearing threshold^7^. In sum, it generally refers to the downward shift of the hearing threshold or loudness discomfort level of the dynamic hearing range due to abnormal auditory gain. People with low loudness discomfort levels may show slower recovery of emotional discomfort caused by noises. Hyperacusis indicates a hypersensitivity to loudness levels where abnormally strong auditory cues occur in most frequency bands. Alternatively, phonophobia or misophonia refers to the avoidance of certain sounds due to discomfort and pain. It is usually accompanied by autonomic nervous system (ANS) responses (cold sweat, palpitation) and limbic system responses (irritation, pain, fear, avoidance)^8^.

Meanwhile, with the recent emergence of various audio-visual reproduction technology, various attempts have been made to promote psychophysiological recovery effect through the reproduced audio-visual environment experience^9–14^. Existing research generally concludes that experiences in natural environments such as forests or urban parks positively affect the psychophysiological recovery of humans. Some studies^12^ have discovered the possibility of potential recovery in natural and urban environments. However, there is insufficient research examining how the difference in individual noise sensitivity affects psychophysiological recovery. Park et al.^15^ examined the difference in recovery response of audio-visual environment experiences due to noise sensitivity. However, they failed to discover a clear difference due to limited stimuli and short experience time. Thus, the correlation between noise sensitivity and psychophysiological response is yet to be found.

Therefore, this study examines how hyperacusis affects emotional and ANS response recovery in various environments (urban and natural) using audio-visual reproduction techniques.

## Methods

### Audio-visual stimuli

**Figure 1:**
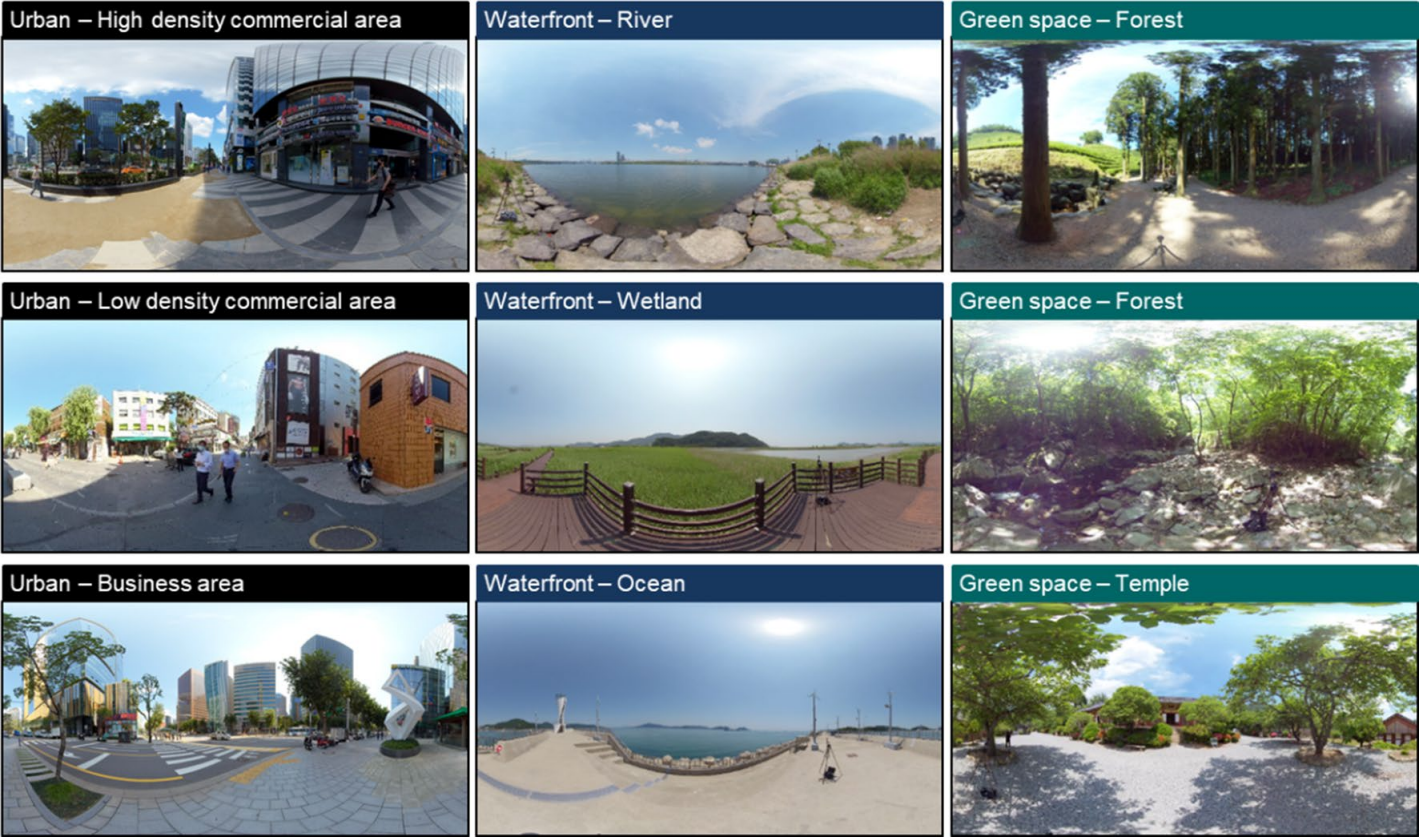
Stitched monoscopic 360-degree view of nine evaluation sites: urban, waterfront, and green areas.

As shown in Fig. 1, we selected nine sites in Korea that represent urban and natural spaces. Typical places where urban residents spend most of their time were selected as urban spaces. These places include high- and low-density commercial and business areas. Natural spaces were classified into waterfront and green spaces. The river, wetland, and ocean were selected as waterfront spaces, while the valley, forest, and temple were included as green spaces. In this study, we attempted to create an environment akin to the actual sites in a lab setting using audio-visual reproduction technology^12,16^. To this end, audio-visual stimuli necessary for creating the audio-visual environment were collected from the selected sites. Measurements were conducted during the daytime in May 2020. Visual information was recorded as monoscopic 360-degree videos using a 6-channel 360-degree camera (Insta 360 pro, Insta 360). Audio information was recorded by connecting a 4-channel ambisonic microphone (SoundField SPS 200, SoundField Ltd.) to a portable sound recorder (Mixpre-6, Sound Devices). A separate calibration microphone was used to calibrate the same sound pressure level as the actual sites in lab settings. All measurements were collected for 3 min at 1.6 m, human eye level from a fixed position.

### Reproduction of audio-visual environment

In order to mimic a measurement condition similar to real life within the laboratory environment proposed in this study, monoscopic 360 video and stereoscopic sound technology was used^17–19^. In a previous study^17^, when monoscopic videos and first-order ambisonic (FOA)-tracked binaural sound were provided, it was confirmed that sufficient fidelity can be obtained because it is possible to obtain a response result that is almost similar to the soundscape evaluation in real space. Therefore, the same audio-visual evaluation environment was implemented in this study as well. The audio-visual environment was created using Unity 3D software (version 2019.4.13f1, https://unity.com/download), and visual information was obtained by stitching 6-channel videos into one 360-degree video. Next, we obtained audio information by converting the A-format FOA sound source into B-format FOA. Subsequently, we down-mixed it into two channels using a spatial audio software development kit (version 1.0.0, https://resonance-audio.github.io/resonance-audio/) that is built in Unity software, creating 3 min of audio-visual stimulation sound source for each point. For the edited audio-visual information, visual and audio information was respectively provided through a head-mounted display (HMD; VIVE Pro, HTC) and open-type headphones (HD-650, Sennheiser). The direction of sound from the head rotation was implemented in real time using the embedded head-tracker on an HMD. Moreover, the sound pressure level of the sound sources played on headphones was adjusted to be equivalent to that of the sound sources recorded using the calibration microphone.

### Experimental design

#### Procedure

**Figure 2:**
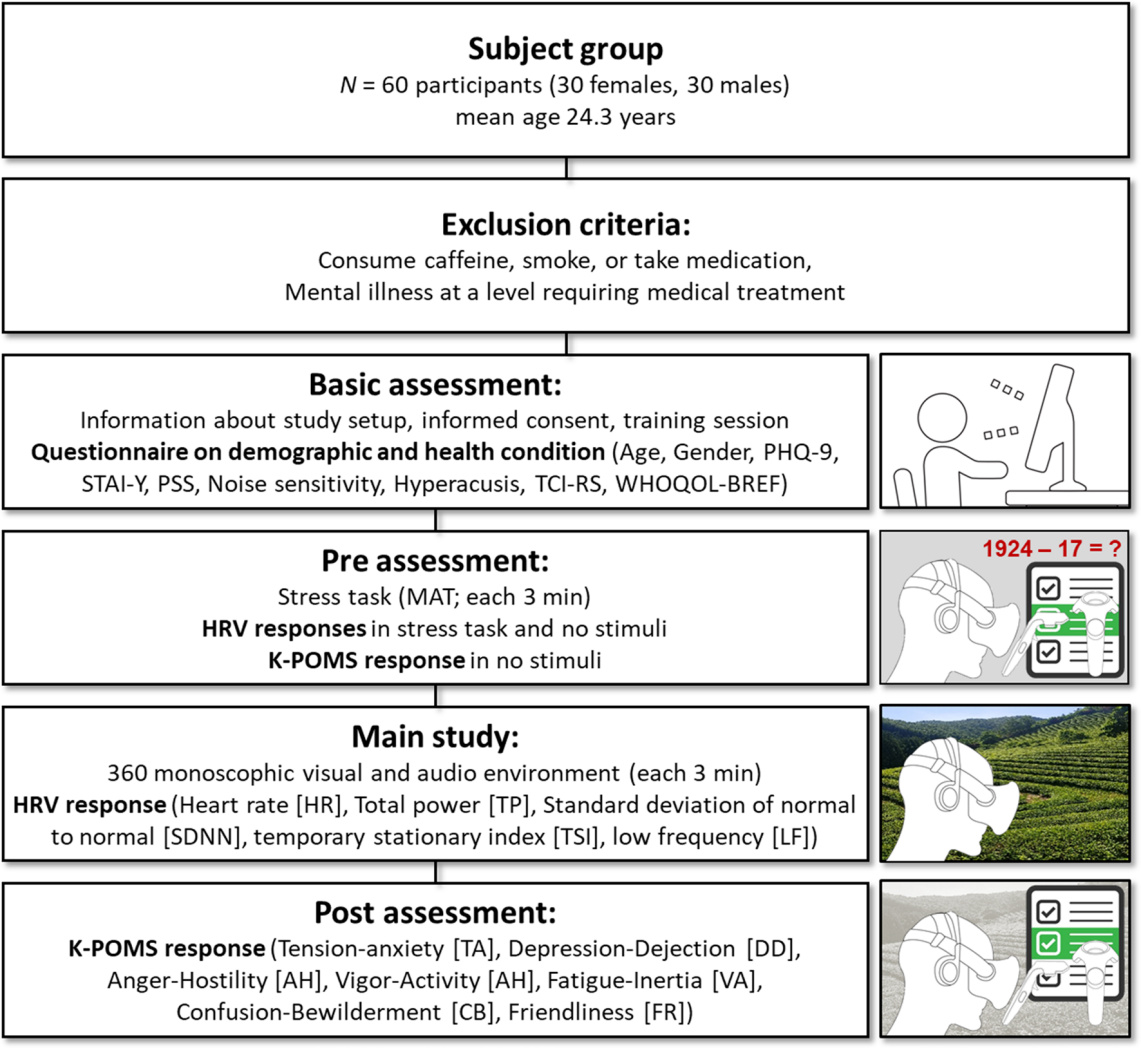
Study procedure. Patient Health Questionnaire (PHQ-9), State-trait Anxiety Inventory (STAI-Y), Perceived Stress Scale (PSS), Temperament and Character Inventory-Revised Short Version (TCI-RS), World Health Organization Quality of Life Questionnaire (WHOQOL-BREF), Mental Arithmetic Tasks (MAT), Heart Rate Variability (HRV), Korean edition of Profile of Mood States (K-POMS).

As shown in Fig. 2, study participants were people with mild depression, stress, and anxiety. In this case, this study aimed to investigate the difference in soundscape recovery response according to noise sensitivity. Therefore, participants were people who demonstrated a desire for emotional recovery and exhibited mild symptoms of emotional disease, which are likely to have a better recovery effect. Whether these criteria were met was judged through a psychiatric diagnosis based on a verified questionnaire scale. Consequently, none of the participants were considered to be at significant clinical risk.

G*Power version 3.1.9.4 (https://www.psychologie.hhu.de/arbeitsgruppen/allgemeine-psychologie-und-arbeitspsychologie/gpower) was used to calculate the study’s sample size. To verify the mean response difference of the noise sensitivity groups, with an effect size of 0.8, a significance level of 0.05, and a power level of 0.8, as determined with the t-test, it is assumed that at least 26 participants in each group and a total of 52 participants are required. To minimize variations in responses, participants were limited to students enrolled in a university or graduate school who had a routine lifestyle with similar daily patterns (mean age = 24.3, standard deviatio*n* = 2.4). The assessment included 60 participants with normal levels of sight and hearing.

Participants were informed regarding the purpose of this study and the questionnaire prior to the beginning of the experiment. This was done to ensure that the participants sufficiently understood the meaning of the questionnaire and its items. The research proposal was approved by the Institutional Review Board as an ethical procedure (HYUIRB-202010-011). The protocol was performed in accordance with the relevant guidelines and regulations. Informed written consent was also received from each participant. Participants were explained how their responses would be used, processed, and stored.

We recommended that they sleep and rest the day before the experiment. They were also not allowed to consume caffeine, smoke, or take any medication that may change their physiological response the day of and before the experiment. On the day of the experiment, each participant’s condition was confirmed through an oral interview, and we found none who exhibited withdrawal symptoms. Participants with sleep and health problems were excluded from the pre-assessment phase. This study was conducted in winter and hence most participants were wearing thick coats. Since the room temperature was maintained at about 24 °C, it was judged that it could be hot or sweaty when participating in the experiment while wearing a coat, and that these factors may cause bias in heart rate variability (HRV) or questionnaire responses. Therefore, with the participants’ consent, the evaluation could be performed in light clothing by taking off the coat.

A simple training session was included to allow those unfamiliar with an HMD device to adjust to the environment. The training session was structured to show audio-visual stimulus sample from a space other than the stimulus used in this experiment and to elicit essential functions required for the experiment, such as response selection and response submission, using a controller while showing part of the questionnaire. Forest environment stimulus from green land, which was different from the nine stimuli used in the experiment, was used as the sample stimulus. This ensured that the participants were sufficiently accustomed to the HMD equipment to preclude any inconvenience during the experiment.

Before the audio-visual assessment, participants put on HRV-measuring hardware, HMD, and headphones to allow the calibration of each device. To set the baseline (reference) standard, HRV responses were collected for 3 min each in the state of stress task and no stimuli. In addition, the Korean edition of Profile of Mood States (K-POMS) responses were collected in the state of no stimuli. Next, HRV responses were collected for 3 min each in the state of stress task and audio-visual stimuli in the main experiment. After the stimuli experience, K-POMS responses were collected. Considering participant fatigue due to the experiment’s length, each participant was exposed to three stimuli (one for the function of each space) randomly selected out of nine. In other words, each participant experienced one urban, waterfront, and green space. Each stimulus was presented to the participants for 3 min. They were provided breaks during the experiment, as needed, to minimize their fatigue or discomfort. All survey responses were recorded using the controller in the audio-visual environment.

#### Questionnaires

The questionnaire consisted of five parts. (1) Demographic information such as gender and age of participants was collected. (2) To examine sound sensitivity and the usual health state, we included nine items of the patient health questionnaire (PHQ-9), 20 items of the state-trait anxiety inventory (STAI-Y), ten items of the perceived stress scale (PSS), 21 items on noise sensitivity^20^, and 14 items on hyperacusis^21^. (3) To assess individuals’ usual personality and temperament, 140 items of the Temperament and Character Inventory-Revised Short Version (TCI-RS)^22^ were added. (4) To examine life satisfaction, 26 items of the World Health Organization Quality of Life Questionnaire (WHOQOL-BREF)^23^ were included. (5) 65 items of the K-POMS^24^ were included to examine the psychological recovery response to each audio-visual environment.

#### Heart rate variability responses

HRV was measured to investigate participants’ physiological responses during the audio-visual environment experience. When the experimenter presented the audio-visual stimuli to the participant, synchronization was performed by simultaneously pressing the stimulus provision button (keyboard) and the HRV measurement button (start button on the HRV hardware) at every stimulus provision by the researcher. Although the experimenter tried to press both the buttons simultaneously, there exists the possibility of a maximum one-second time difference occurring between each press of the button. As this study examined the participants’ average psychophysiological response over 3 min, the experiment was performed under the assumption that this time difference would not have a significant impact on the interpretation of our results.

HRV shows the periodic change in heart rate over time and is closely related to the interaction between sympathetic and parasympathetic nerves. SA-3000NEW (Medicore, Korea) was used for measurement, and the sensor was attached to the inner side of the participants’ wrists and ankles to measure their HRV for 3 min. Five indicators were selected, and the results were quantified into means of 3 min: (1) heart rate (HR) indicating the average heartbeat for 1 min, (2) total power indicating the vitality of ANS, (3) standard deviation of normal to normal (SDNN) indicating stress resistance, (4) temporary stationarity index representing stress, and (5) low frequency as an index for sympathetic nerve activity and indicated fatigue. The HRV response was obtained by calculating the relative difference (%)^25^ instead of the absolute value of the measures, using the formula “(raw value − stress-state value)/stress-state value $\times$ 100”. Here, physiological responses when experiencing audio-visual stimuli were used as raw values. Errors in physiological responses among individual participants were minimized through this normalization process.

#### Stress task

We provided computerized mental arithmetic tasks to induce stress in participants before the audio-visual environment experience. Mental subtraction problems were shown for 1 s to the participants in the audio-visual environment, which they were asked to solve in 3 s. If answered incorrectly, they were to repeat the same problem from the beginning, thereby inducing a stress reaction. In addition, participants were asked to perform these tasks with their eyes open, with the exception of natural blinking. However, there were limitations in ensuring that the participants kept their eyes open despite the request of the researcher.

### Data analysis

Responses of 20 participants were obtained at each assessment site; thus, 180 results (9 sites $\times$ 20 responses) were collected for each assessment item. The following analyses were conducted using SPSS Statistics (IBM, version 25). All responses were tested for normality (Shaprio-Wilk and Kolmogorov–Smirnov) and homoscedasticity (Levene). The data satisfied normality, and thus parametric statistics were conducted. Analysis of variance was conducted to examine whether there is a statistically significant difference in K-POMS and HRV responses depending on the function of the space. K-means clustering was used to classify the participants by individual health state, including hyperacusis. Additionally, a t-test was performed to determine the significance of the difference in psychophysiological responses between the groups. To assess the difference in the responses of the groups according to sensitivity, the effect size (Cohen’s d) was calculated.

## Results

As shown in Table 1, most participants showed high stress levels at 19 points or higher. Additionally, some showed high depression at ten points or higher, proving that the participants recruited in this study usually exhibited an active need for emotional recovery. There was a wide distribution of noise sensitivity, personality, temperament, and life satisfaction.

**Table 1:** Summary of demographical and health conditions of participants.

Parameter	Mean (SD)	Min	Max
Age (number)	24.3 (2.4)	21	30
*Male (30)*	25.5 (2.3)	21	30
*Female (30)*	23.2 (1.9)	21	28
**Health condition (number)**			
Patient health questionnaire (PHQ-9)	3.9 (3.2)	0	14
*PHQ-9 0–4 (39)*	2.0 (1.5)	0	4
*PHQ-9 5–9 (17)*	6.2 (1.4)	5	9
*PHQ-9 10–19 (4)*	11.8 (1.7)	10	14
State-trait anxiety inventory (STAI-Y)	20.3 (5.3)	8	34
Perceived stress scale (PSS)	21.7 (4.2)	12	30
*PSS 0–12 (3)*	12.0 (0.0)	12	12
*PSS 13–15 (1)*	14 (0.0)	14	14
*PSS 16–18 (7)*	17.3 (0.0)	16	18
*PSS 19–40 (49)*	23.0 (3.1)	19	30
Noise sensitivity	79.1 (16.1)	45	112
Hyperacusis	11.4 (6.6)	1	26
**Temperament and character dimension**			
Novelty Seeking (NS)	58.5 (10.0)	40.0	88.0
Harm avoidance (HA)	49.4 (12.2)	22.0	73.0
Reward dependence (RD)	54.9 (13.9)	23.0	89.0
Persistence (P)	51.4 (10.7)	31.0	75.0
Self-directiveness (SD)	51.6 (11.2)	17.0	77.0
Cooperativeness (C)	49.8 (13.4)	1.0	77.0
Self-transcendence (ST)	46.8 (9.2)	29.0	70.0
**Quality of life (WHOQOL-BREF)**			
Total score	68.9 (8.9)	42.8	86.6

Cluster analysis was conducted to classify 60 participants based on their health state. We used the K-means clustering method known to be efficient and applicable to various types of data. Five indicators (PHQ-9, STAI-Y, PSS, noise sensitivity, hyperacusis) were used as independent variables. Since there were five independent variables, Minkowski distance was used, which is suitable for at least two-dimensional data as a subjective measure for similarity among participant responses.

It is necessary to determine the number of clusters in advance for K-means clustering, and the results vary depending on that number. Thus, the verification process was conducted using “NbClust” package^26^ provided by R language to set the optimal number of clusters. As shown in Table 2, the number of clusters was determined based on 26 indices. As a result, clusters were classified into two clusters recommended by 12 out of 26 indices. Thus, 28 participants were classified into Cluster 1, and 32 into Cluster 2.

**Table 2:** Twenty-six index values for determining the optimal number of clusters.

Methods	Index	Year	Authors	N	Index value
Maximum/minimum value of the index	CH	1974	Calinski and Harabasz	2	76.7
	Dunn	1974	Dunni	4	0.2
	McClain	1975	McClain and Rao	2	0.5
	Cindex	1976	Hubert and Levin	10	0.4
	DB	1979	Davies and Bouldin	2	0.9
	Ptbiserial	1980	Millian	2	0.6
	CCC	1983	Sarle	2	19.7
	Silhouette	1987	Rowsseeuw and Lai	2	0.4
	KL	1988	Krzanowski and Lai	9	48.5
	SDindex	2000	Halkidi et al	2	0.2
	SDbw	2001	Halkidi and Vazirgiannis	10	0.1
Maximum difference between hierarchy levels of the index	Ball	1965	Ball and Hall	3	2196.9
	Friedman	1967	Friedman and Bubin	0	0.0
	Scott	1971	Scott and Symons	4	45.3
	Hartigan	1975	Hartigan	4	8.7
	Ratkowsky	1978	Ratkowsky and Lance	2	0.3
	TrCovW	1985	Milligan and Cooper	3	2.4 $\times$ 10^6^
Etc	Rubin	1967	Friedman and Rubin	9	−8.6
	Beale	1969	Beale	2	−0.5
	Marriot	1969	Marriot	2	4.6 $\times$ 10^15^
	Frey	1972	Frey and Van Groenewoud	4	1.4
	Pseudot2	1973	Duda and Hart	2	−7.0
	Duda	1973	Duda and Hart	2	1.2
	TraceW	1985	Miligan and Cooper	10	269.6
	Hubert	1985	Hubgert and Arobie	4	898.2
	Dindex	2000	Lebart et al	0	0.0

**Figure 3:**
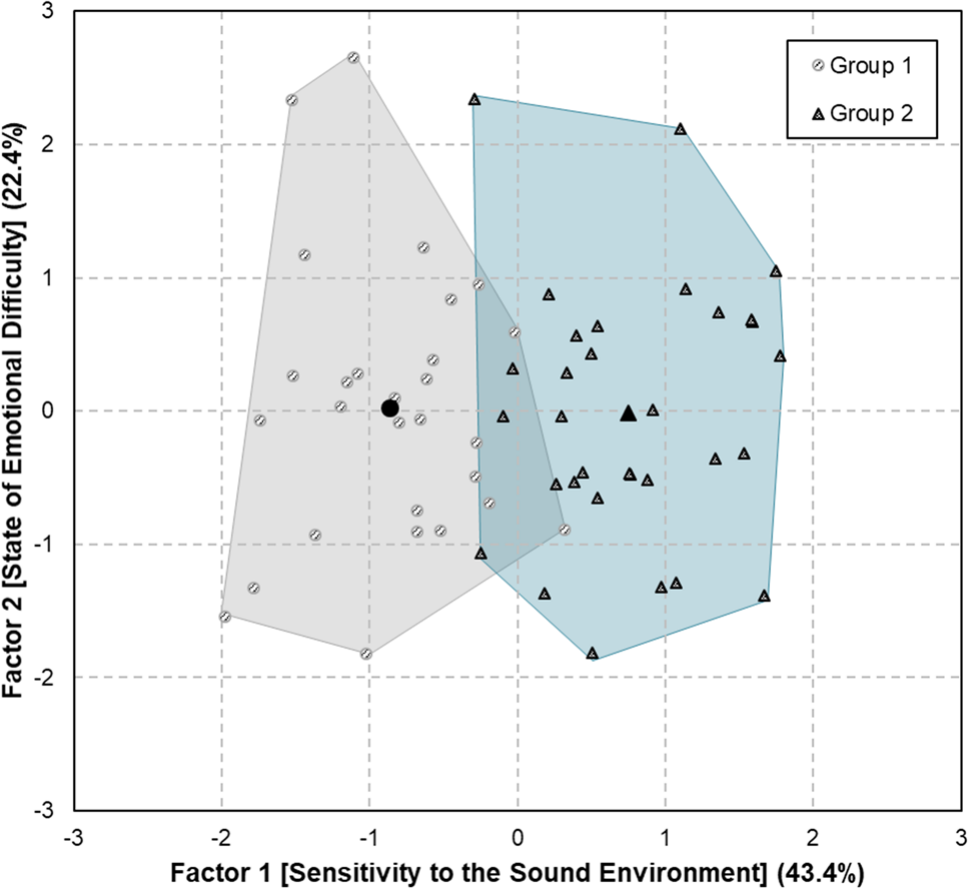
Cluster analysis results based on health conditions. Groups 1 and 2 represent low and high noise-sensitivity, respectively.

Principle component analysis was conducted on the five cases of health data, and the distribution of Clusters 1 and 2 is provided in Fig. 3 based on the results. Varimax rotation was applied here. The statistical model was significant with Kaiser–Meyer–Olkin’s measure of sampling adequacy below 0.564 and Bartlett’s test of sphericity below 0.05. Factor 1 had an explanatory power of 43.4% and showed high sensitivity (loading = 0.919), hyperacusis (loading = 0.854), and STAI-Y (loading = −0.173), thereby representing sensitivity to the sound environment. Factor 2 had an explanatory power of 22.4% and was highly correlated with PHQ-9 (loading = 0.907) and PSS (loading = 0.856), thereby representing the state of emotional difficulty. As a result, Groups 1 and 2 were clearly divided based on the standard of factor 1.

Table 3 compares the differences in demographic and health conditions between the two groups based on the previously examined standard. A t-test was performed to test the statistical significance of the mean difference between the two groups; this is presented in Table 3. As a result, characteristic values excluding noise sensitivity and hypersensitivity (gender, age, depression, anxiety, stress, personality, life satisfaction) did not show a significant difference. Cohen’s d, the effect size, of the mean difference in noise sensitivity and hypersensitivity between the two groups was high at 3.26 and 1.42, respectively. Therefore, Group 1 was relatively less sensitive to sound than Group 2, and they can be referred to as the low and high noise-sensitive group, respectively.

**Table 3:** Difference of demographic and health condition according to different groups.

Methods	Group 1	Group 2	t	*P*-value	95% CI of the diff		Effect size	Power
					Lower	Upper		
Gender	Number	Number						
Male	16	14						
Female	12	18						
Year	Mean (SD)	Mean (SD)						
Age	24.1 (2.5)	24.6 (2.3)	−0.80	.43	−1.72	0.74	0.21	0.12
**Health condition**								
PHQ	3.4 (3.4)	4.2 (3.0)	−0.96	.34	−2.45	0.87	0.25	0.16
STAI-Y	21.2 (4.7)	19.5 (5.8)	1.20	.24	−1.11	4.41	0.32	0.22
PSS	21.0 (4.0)	22.2 (4.3)	0.26	.26	−3.37	0.94	0.29	0.20
Noise sensitivity	64.5 (8.6)	91.8 (8.4)	−12.45	< .01	−31.67	−22.89	3.26	1.00
Hyperacusis	7.4 (5.0)	14.9 (5.7)	−5.40	< .01	−10.35	−4.75	1.42	1.00
**Temperament and character dimension**								
Novelty seeking	58.4 (10.1)	58.7 (10.1)	−0.13	.90	−5.54	4.88	0.03	0.05
Harm Avoidance	46.6 (10.5)	51.9 (13.2)	−1.70	.10	−11.48	0.95	0.45	0.39
Reward dependence	53.7 (13.1)	55.9 (14.8)	−0.60	.55	−9.41	5.09	0.16	0.09
Persistence	53.1 (11.4)	49.9 (9.9)	1.19	.24	−2.23	8.77	0.31	0.22
Self-directiveness	54.1 (10.1)	49.5 (11.9)	1.61	.11	−1.13	10.34	0.42	0.35
Cooperativeness	50.7 (13.2)	49.1 (13.7)	0.46	.64	−5.36	8.59	0.12	0.07
Self-transcendence	46.1 (9.4)	47.4 (9.1)	−0.53	.60	−6.06	3.52	0.14	0.08
**Quality of life (WHOQOL-BREF)**								
Total score	71.0 (8.3)	67.1 (9.2)	1.70	.10	−0.70	8.41	0.45	0.39

**Figure 4:**
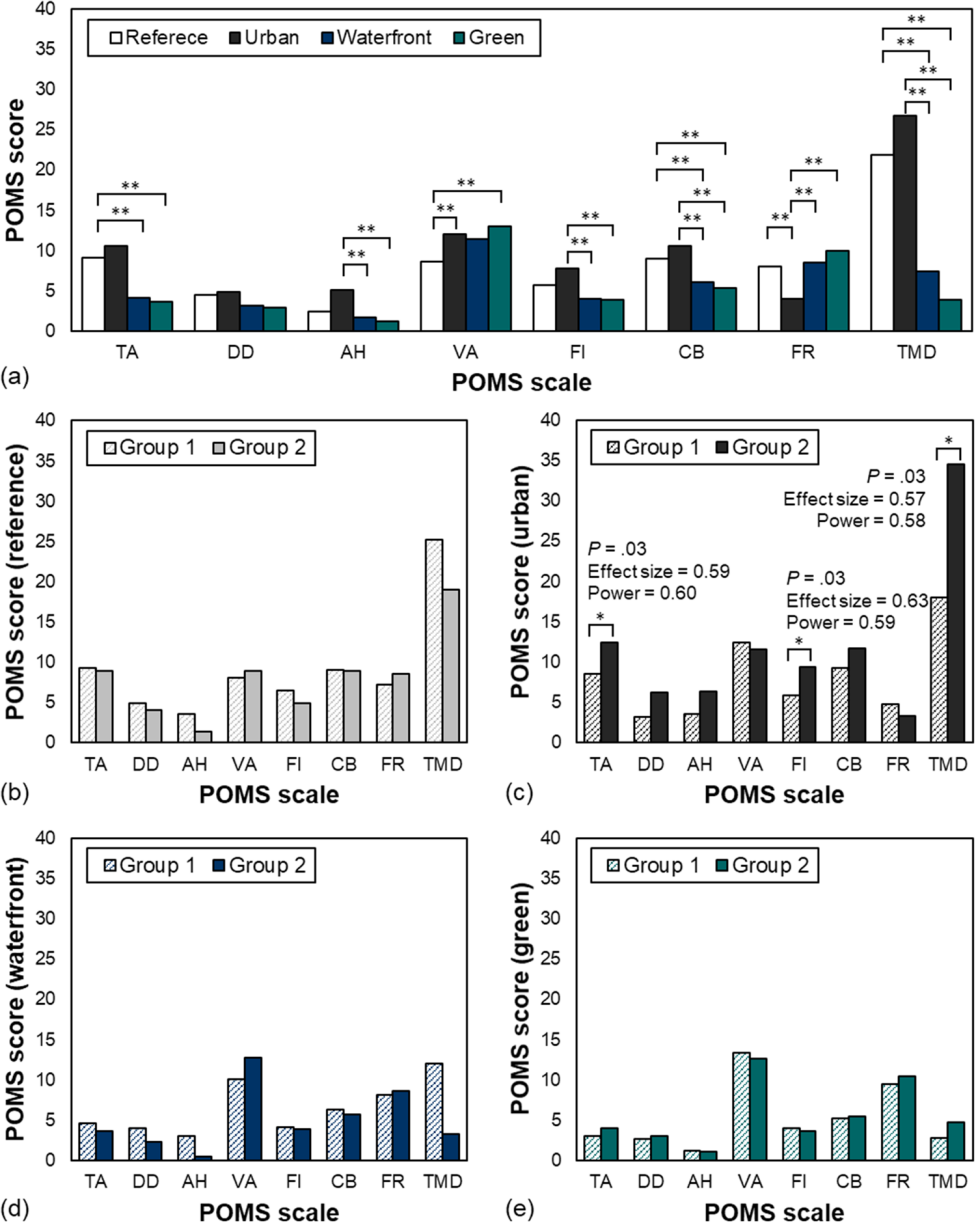
Differences in the Korean edition of Profile of Mood States (K-POMS) before and during audio-visual stimuli experience. (**a**) K-POMS difference according to space function, (**b**) K-POMS difference in reference with different groups, (**c**) K-POMS difference in urban with different groups, (**d**) K-POMS difference in waterfront with different groups, (**e**) K-POMS difference in green with different groups. Tension-Anxiety (TA), Depression-Dejection (DD), Anger-Hostility (AH), Vigor-Activity (VA), Fatigue-Inertia (FI), Confusion-Bewilderment (CB), Friendliness (FR), Total Mood Disturbance (TMD; **P-value < .001, *P-value < .05).

Emotional recovery responses to audio-visual stimuli experience were examined through K-POMS, and the results are as shown in Fig. 4. They were classified into eight measures: Tension-Anxiety (TA), Depression-Dejection (DD), Anger-Hostility (AH), Vigor-Activity (VA), Fatigue-Inertia (FI), Confusion-Bewilderment (CB), Friendliness (FR), and Total Mood Disturbance (TMD). Figure 4a shows that emotional changes depend on the function of space. Compared to the reference value, negative emotional responses increased slightly overall in the urban environment, while they decreased with statistical significance in the natural environment. In terms of emotional change in the reference and the city, there was a slight increase in negative responses, but not with statistical significance. This is because being mostly familiar with the city, participants did not exhibit much resistance to or negative views about the urban environment. As for the emotional difference between urban and natural environments, negative emotional responses such as AH, FI, CB, and TMD were lower in nature, indicating that the natural environment created in an audio-visual environment had a psychologically positive effect on the participants.

The results of analyzing the two groups depending on noise sensitivity are presented in Fig. 4b–e by the function of space. As shown in Fig. 4b, there was a significant difference in emotional responses between the two groups in the city. Group 2 (high noise-sensitive group) showed higher TA, FI, and TMD than Group 1 (low noise-sensitive group) by 3.97, 0.52, and 16.50, respectively. In the city, Cohen’s d of the mean difference in TA, FI, and TMD between the groups was high at 0.59, 0.63, and 0.57, respectively. *Based on Cohen’s criteria, the effect size was medium and significant.* On the other hand, there was no significant difference in waterfront and green areas between the two groups in the natural environment.

**Figure 5:**
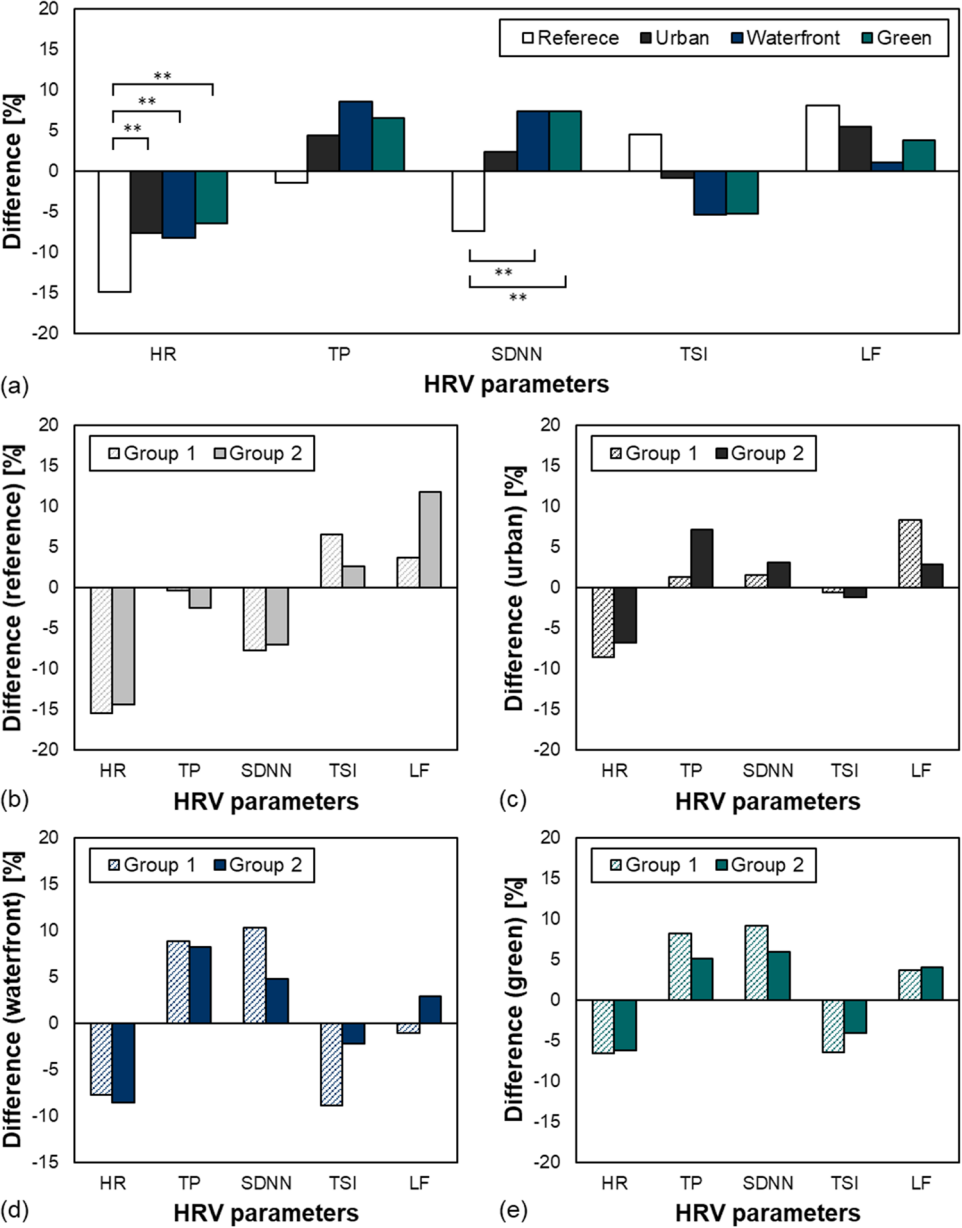
Differences in the heart rate variability (HRV) before and during audio-visual stimuli experience. (**a**) HRV difference according to space function, (**b**) HRV difference in reference with different groups, (**c**) HRV difference in urban with different groups, (**d**) HRV difference in waterfront with different groups, (**e**) HRV difference in green with different groups. Heart Rate (HR), Total Power (TP), Standard Deviation of Normal to Normal (SDNN), Temporary Stationary Index (TSI), Low Frequency (LF; **P-value < .001).

Physiological recovery responses through audio-visual stimuli experience were examined using HRV, and the results are shown in Fig. 5. First, Fig. 5a shows the changes in HRV depending on the function of space, akin to the results of emotional responses previously examined. The results showed that HR increased by 7.24, 6.64, and 8.42% each from the reference in all urban, waterfront, and green spaces, respectively, with statistical significance. Moreover, SDNN increased by 14.70 and 14.74% in waterfront and green spaces, respectively, than the reference with statistical significance. SDNN refers to how irregular and complicated HRV was during recording time. Irregular HR indicates that the homeostatic mechanism of the ANS is working properly, and that the coping skills for various stressors are being improved. Therefore, similar to the emotional responses that were previously examined, we found that the natural environment created in the audio-visual environment can induce a physiological recovery effect in the participants even in their physiological responses. Next, the results of the two groups by noise sensitivity are provided in Fig. 5b–e depending on the function of space. Unlike the emotional responses, there was no significant difference between the two groups in physiological responses.

## Discussion

This study examined whether noise sensitivity or hyperacusis affects emotional and ANS response recovery through an audio-visual environment experience. First, considering the psychophysiological differences according to the function of the place, the restoration response was higher in the natural as opposed to the urban environment, and there was no significant restoration difference between the waterfront and the green space within the natural environment. However, when comparing psychophysiological responses between groups according to noise sensitivity, the difference could only be confirmed in the urban environment, not the natural environment.

At this time, the cause of the difference in psychophysiological response according to noise sensitivity is analyzed from a pathological point of view. Hyperacusis is a disease that can be explained by integrating multifactorial causes and hypotheses as “abnormal auditory gain.” In other words, abnormally excessive neural excitement occurs compared to the loudness of the sound input^27^. Hyperacusis mostly indicates decreased sound tolerance and sometimes the decline of the hearing threshold, that is, abnormally excellent hearing. Various illnesses can cause the two symptoms, but they can also occur when there is an excessive auditory gain due to a disorder in the auditory gain control mechanism. In this study, the group with relatively high noise sensitivity may have slowed down the emotional recovery due to more neural excitement than recovery due to the natural environment stimuli. Changes in afferent neural plasticity, neurotransmitter, hormones, and olivocochlear efferent control disorder have been studied as the pathological mechanisms of the central auditory system related to hyperacusis, leading to an abnormal increase in auditory gain (number of neural signals generated). The sounds in the natural environment as the common relaxation stimulus in excessive signals are correlated to the increase in overall neural signals, thereby contributing to negative emotions rather than the relaxation effect.

The same environmental stimuli can be provided in a controlled environment by using the audio-visual environment. The group with high noise sensitivity in previous studies on indoor noise sensitivity reported higher annoyance when the sound came from the outside^28^. For the group with low noise sensitivity, external noise has a “masking effect” over internal noise. In contrast, external noise may have a “moderating effect” that increases discomfort toward internal noise for the group with high noise sensitivity. For the participants sensitive to noise, recognition of the negative acoustic environment due to exposure to external noise may have affected the recognition of internal noise^28^. This study assessed emotional recovery responses to the audio-visual stimuli experienced by dividing participants into a control and a hyperacusis group. The latter showed significantly high TA, FI, and TMD in the urban environment. Moreover, an effect size greater than 0.5 suggested high practical significance. Thus, there was a significant difference in the emotional responses between the two groups but only in the urban environment. The emotional difference due to noise sensitivity may have been more apparent in the urban than the natural environment that is relatively fixed since the city has various noise sources (road traffic noise, construction noise, noise caused by human activities, music, etc.). This is in line with the fact that the high noise sensitivity group showed a higher annoyance response to an external noise source in the preceding study. Therefore, clustering based on noise sensitivity and hyperacusis in this study may be a significant method in examining the emotional differences in urban environments.

The limitations of this study are as follows. First, only subjective assessment results were used for clustering based on noise sensitivity. Second, while the participants were classified by noise sensitivity, they were not patient groups with hyperacusis. It is necessary to check whether the results of this study can be reproduced using clinical patients with hyperacusis. Third, no difference was found between the two groups in the HRV tests to check the actual physiological change. This may be because HRV measurement failed to closely reflect changes due to the relatively short experiment time. In the process of measuring the physiological response, the researcher simultaneously pressed HRV hardware button and generated stimulus. However, the minimal differences in the time may have affected the physiological responses. Thus, it would be necessary to supplement the methods for triggers in subsequent studies. It is also necessary to track and verify the effect of the recovery environment over a long period. In this study, although psychophysiological differences in noise sensitivity according to differences in individual characteristics such as personality and life satisfaction could not be confirmed, it is necessary to conduct in-depth research on the influence of individual characteristics through follow-up studies. In addition, there were limitations regarding the participant group composition that only included the urban population and individuals in their twenties; thus, caution should be exercised in drawing generalized conclusions from this study’s results. Furthermore, despite the advancement of virtual reality technology, visual fidelity is still somewhat lacking compared to the real space due to image quality and latency problems, so comparison with the real space is required in the future. In addition, it must be considered that familiarity with hardware devices, such as HMD, can influence the results of our experiment.

Nonetheless, this study provided stimuli using the controlled audio-visual environment, and assessed the emotional states such as anxiety, subjective stress, and depression in advance, showing no difference in emotional pathology between groups. Negative emotions such as depression and anxiety are both the cause and effect of hyperacusis. However, we controlled this in advance and were able to check the group characteristics based on pure noise sensitivity differences. Further research is needed on clinical patients with hyperacusis to determine whether actual noise sensitivity affects emotional and environmental recovery. Moreover, it is necessary to verify whether this short-term emotional recovery effect is maintained continuously through long-term tracking and monitoring.

This study examined how experiences of urban and natural environments affect the recovery of emotional and ANS responses of people with mild depression, anxiety, and stress. Overall, the participants showed higher emotional and ANS recovery responses in the natural as opposed to the urban environment. Moreover, the recovery difference was not significant in the waterfront and green spaces. There was a difference in the psychophysiological response between the groups depending on noise sensitivity but only in the urban environment. Therefore, noise sensitivity was proven to be a critical factor that must be considered when investigating emotional responses in the urban environment. This finding can be used as basic data for creating personalized audio-visual recovery contents considering the individual characteristic of noise sensitivity and in developing various contents to promote psychophysiological recovery of humans through the audio-visual environment experience.

## Supplementary Information

Supplementary Information.

## Data Availability

Supporting data will be made available to editorial board members and referees at the time of submission for the purposes of evaluating the manuscript.

## References

[1] Ryu JK, Jeon JY (2011). Influence of noise sensitivity on annoyance of indoor and outdoor noises in residential buildings. Appl. Acoust..

[2] Zimmer K, Ellermeier W (1999). Psychometric properties of four measures of noise sensitivity: A comparison. J. Environ. Psychol..

[3] Ryu, J-K. & Jeon, J-Y. The effects of sensitivity on subjective responses to residential noises. In: *Proceedings of the KSNVE Annual Spring Conference*, pp. 363–366 (2005).

[4] Anari M, Axelsson A, Eliasson A, Magnusson L (1999). Hypersensitivity to sound: Questionnaire data, audiometry and classification. Scand. Audiol..

[5] Park SN (2002). Hyperacusis in patients with tinnitus-audiometrical evaluation and clinical characterization. Korean J. Otolaryngol. Head Neck Surg..

[6] Vernon JA (1987). Pathophysiology of tinnitus: a special case—hyperacusis and a proposed treatment. Am. J. Otol..

[7] Yuen, H-W., Eikelboom, R. H. & Atlas, M. D. Auditory manifestations of superior semicircular canal dehiscence. *Otol. Neurotol*. **30,** 280–285 (2009). 10.1097/mao.0b013e31819d895e 19326497

[8] Jastreboff, P. J. Decreased sound tolerance in *Tinnitus: Theory and Management* (ed. Snow, J. B.) 8–15 (McGraw Hill Education, 2004).

[9] Annerstedt M (2013). Inducing physiological stress recovery with sounds of nature in a virtual reality forest—Results from a pilot study. Physiol. Behav..

[10] Gao T, Zhang T, Zhu L, Gao Y, Qiu L (2019). Exploring psychophysiological restoration and individual preference in the different environments based on virtual reality. Int. J. Environ. Res..

[11] Hedblom M (2019). Reduction of physiological stress by urban green space in a multisensory virtual experiment. Sci. Rep..

[12] Jeon JY, Jo HI, Lee K (2021). Potential restorative effects of urban soundscapes: Personality traits, temperament, and perceptions of VR urban environments. Landsc. Urban Plan..

[13] Mattila O (2020). Restoration in a virtual reality forest environment. Comput. Hum. Behav..

[14] Yu, C-P., Lee, H-Y. & Luo, X-Y. The effect of virtual reality forest and urban environments on physiological and psychological responses. *Urban For. Urban Green*. **35,** 106–114 (2018).

[15] Park SH, Lee PJ, Jung T, Swenson A (2020). Effects of the aural and visual experience on psycho-physiological recovery in urban and rural environments. Appl. Acoust..

[16] Jo HI, Jeon JY (2021). Overall environmental assessment in urban parks: Modelling audio-visual interaction with a structural equation model based on soundscape and landscape indices. Build. Environ..

[17] Hong JY (2019). Quality assessment of acoustic environment reproduction methods for cinematic virtual reality in soundscape applications. Build. Environ..

[18] Jeon JY, Jo HI (2020). Effects of audio-visual interactions on soundscape and landscape perception and their influence on satisfaction with the urban environment. Build. Environ..

[19] Jo HI, Jeon JY (2020). Effect of the appropriateness of sound environment on urban soundscape assessment. Build. Environ..

[20] Weinstein ND (1978). Individual differences in reactions to noise: a longitudinal study in a college dormitory. J. Appl. Psychol..

[21] Khalfa S (2002). Psychometric normalization of a hyperacusis questionnaire. ORL J. Otorhinolaryngol. Relat. Spec..

[22] Min, B., Oh, H. & Lee, J. Temperament and character inventory-family manual. *Seoul Maumsarang*. 6–14 (2007).

[23] Min SK, Lee CI, Kim KI, Suh SY, Kim DK (2000). Development of Korean version of WHO quality of life scale abbreviated version (WHOQOL-BREF). J. Korean Neuropsychiatr. Assoc..

[24] Kim, E-J., Lee, S-I., Jeong, D-U., Shin, M-S. & Yoon, I-Y. Standardization and reliability and validity of the Korean edition of Profile of Mood States (K-POMS). *Sleep Med. Psychophysiol*. **10,** 39–51 (2003).

[25] Li Z, Kang J (2019). Sensitivity analysis of changes in human physiological indicators observed in soundscapes. Landsc. Urban Plan..

[26] Charrad M, Ghazzali N, Boiteau V, Niknafs A (2014). NbClust: an R package for determining the relevant number of clusters in a data set. J. Stat. Softw..

[27] Diehl PU, Schaette R (2015). Abnormal auditory gain in hyperacusis: investigation with a computational model. Front. Neurol..

[28] Park, S. H., Shin, H-K. & Kim, K. W. VR experiment on indoor noise perception and moderation effects of outdoor sounds, visual environment and noise sensitivity. *Trans. Korean Soc. Noise Vib. Eng*. **31,** 279–288 (2021).

